# Isolation of Cancer-Derived Exosomes Using a Variety of Magnetic Nanostructures: From Fe_3_O_4_ Nanoparticles to Ni Nanowires

**DOI:** 10.3390/nano10091662

**Published:** 2020-08-25

**Authors:** Zohreh Nemati, Mohammad Reza Zamani Kouhpanji, Fang Zhou, Raja Das, Kelly Makielski, Joseph Um, Manh-Huong Phan, Alicia Muela, Mᵃ Luisa Fdez-Gubieda, Rhonda R. Franklin, Bethanie J. H. Stadler, Jaime F. Modiano, Javier Alonso

**Affiliations:** 1 Department of Electrical and Computer Engineering, University of Minnesota, Minneapolis, MN 55455, USA; zaman022@umn.edu (M.R.Z.K.); umxxx023@umn.edu (J.U.); rfrank01@umn.edu (R.R.F.); stadler@umn.edu (B.J.H.S.); 2 Animal Cancer Care and Research Program, University of Minnesota, Saint Paul, MN 55108, USA; makie001@umn.edu (K.M.); modiano@umn.edu (J.F.M.); 3 Masonic Cancer Research Center, University of Minnesota, Minneapolis, MN 55455, USA; 4 Department of Biomedical Engineering, University of Minnesota, Minneapolis, MN 55455, USA; 5 Shepherd Labs, University of Minnesota, Minneapolis, MN 55455, USA; zhoux341@umn.edu; 6 Faculty of Materials Science and Engineering and Phenikaa Institute for Advanced Study (PIAS), Phenikaa University, Hanoi 10000, Vietnam; raja@phenikaa-uni.edu.vn; 7 Phenikaa Research and Technology Institute (PRATI), A & A Green Phoenix Group, 167 Hoang Ngan, Hanoi 10000, Vietnam; 8 Department of Veterinary Clinical Sciences, College of Veterinary Medicine, University of Minnesota, Saint Paul, MN 55108, USA; 9 Department of Physics, University of South Florida, Tampa, FL 33620, USA; phanm@usf.edu; 10 BCMaterials, Basque Center for Materials, Applications and Nanostructures, UPV/EHU Science Park, 48940 Leioa, Spain; alicia.muela@ehu.eus (A.M.); malu.gubieda@ehu.eus (M.L.F.-G.); 11 Department of Immunology, Microbiology, and Parasitology, University of Basque Country (UPV/EHU), 48940 Leioa, Spain; 12 Department of Electricity and Electronics, University of Basque Country (UPV/EHU), 48940 Leioa, Spain; 13 Department of Chemical Engineering and Materials Science, University of Minnesota, Minneapolis, MN 55455, USA; 14 Center for Immunology, University of Minnesota, Minneapolis, MN 55455, USA; 15 Stem Cell Institute, University of Minnesota, Minneapolis, MN 55455, USA; 16 Department CITIMAC, University of Cantabria (UC), 39005 Santander, Spain

**Keywords:** cancer exosomes, magnetic isolation, nanowires, nanorods, magnetosomes

## Abstract

Isolating and analyzing tumor-derived exosomes (TEX) can provide important information about the state of a tumor, facilitating early diagnosis and prognosis. Since current isolation methods are mostly laborious and expensive, we propose herein a fast and cost-effective method based on a magnetic nanoplatform to isolate TEX. In this work, we have tested our method using three magnetic nanostructures: (i) Ni magnetic nanowires (MNWs) (1500 × 40 nm), (ii) Fe_3_O_4_ nanorods (NRs) (41 × 7 nm), and (iii) Fe_3_O_4_ cube-octahedral magnetosomes (MGs) (45 nm) obtained from magnetotactic bacteria. The magnetic response of these nanostructures has been characterized, and we have followed their internalization inside canine osteosarcoma OSCA-8 cells. An overall depiction has been obtained using a combination of Fluorescence and Scanning Electron Microscopies. In addition, Transmission Electron Microscopy images have shown that the nanostructures, with different signs of degradation, ended up being incorporated in endosomal compartments inside the cells. Small intra-endosomal vesicles that could be precursors for TEX have also been identified. Finally, TEX have been isolated using our magnetic isolation method and analyzed with a Nanoparticle tracking analyzer (NanoSight). We observed that the amount and purity of TEX isolated magnetically with MNWs was higher than with NRs and MGs, and they were close to the results obtained using conventional non-magnetic isolation methods.

## 1. Introduction

Exosomes are extracellular vesicles released by most eukaryotic cells that contain materials (RNA, proteins, etc.) unique to their parent cells [1,2]. They are composed of a lipid bilayer, with a size in the range 30–160 nm, and they typically present a round morphology [3]. In the same way, cancer cells also release circulating tumor exosomes (TEX). Tumor cells use TEX to communicate with local and distant environments that play key roles in the tumor growth and metastasis [4]. Despite the progress in the fight against this disease, cancer still remains one of the major causes of death in the world [5]. In many cases, the survival rate is rather poor, due to the lack of early diagnosis and personalized treatment. Since TEX package materials that can reveal the state of the tumor cells, they are currently being used as diagnostic and prognostic biomarkers for various cancers [6,7].

In order to isolate TEX from culture media, different techniques are currently being employed (immunoaffinity, ultracentrifugation, precipitation, etc. [8]). Unfortunately, most of these techniques require expensive equipment and/or a laborious process. Therefore, there is a pressing need for developing a low-cost, fast, and efficient TEX isolation technique. In a previous work, we proved that TEX can be isolated from culture media by using a novel method based on the magnetic separation of TEX using magnetic nanowires (MNWs) [9]. This method is essentially involved in feeding the cancer cells with MNWs, which were consequently encapsulated into endosomes inside the cells and then released to the culture medium attached to TEX. Therefore, by using a simple magnet, we managed to effectively isolate those TEX-containing MNWs. Finally, we showed that the TEX isolated with our method presented similar characteristics (size distribution, purity) to those isolated using conventional methods. These results validated our magnetic isolation method, proving that it can consistently provide relatively high TEX yields in a low-cost and fast manner [9]. However, we found some shortcomings within this method: the progressive degradation (surface damage, fragmentation, etc.) of the MNWs implied that their magnetic response was weakened, thus hindering their magnetic isolation capacity; the magnetically isolated TEX were quantified through indirect methods, but no direct observation of the TEX was attained. In order to tackle these and other emerging questions, and to further reinforce the validity of our magnetic isolation method, in this work, we have employed three different types of magnetic nanostructures for TEX isolation, covering a variety of sizes, shapes, compositions, and magnetic responses. These are: (i) Ni MNWs synthesized through electrodeposition, (ii) Fe_3_O_4_ nanorods (NRs) prepared by the hydrothermal method, and finally (iii) Fe_3_O_4_ magnetic nanoparticles biomineralized by magnetotactic bacteria, which are called “magnetosomes” (MGs). Although our magnetic isolation method was originally devised to be used with MNWs, magnetite/maghemite-based nanostructures present better biocompatibility, and currently, they are the only ones approved by the U.S. Food and Drug Administration (FDA) and the European Medicines Agency (EMA) for use in humans. Therefore, we have tried to test the viability of these Fe_3_O_4_-based nanostructures for the magnetic isolation of TEX. In addition, magnetite-based nanoparticles can eventually open the possibility of using these TEX + nanoparticles systems for cancer theranostics, such as in MRI, drug delivery, and magnetic hyperthermia [10,11,12,13,14].

In this work, after a careful characterization of the structural and magnetic properties of these three different types of magnetic nanostructures, we have performed their internalization inside the OSCA-8 cells, analyzed the particular differences observed in each case, and studied the isolated TEX using electron microscopy. Finally, we have studied and compared their magnetic isolation efficiency and the size distribution of the TEX isolated using Nanosight [15].

## 2. Materials and Methods

### 2.1. Materials

All materials were used as received. Nickel (II) sulfate hexahydrate (NiSO_4_·6H_2_O), boric acid (H_3_BO_3_), and fluorescein polyethylene glycol (PEG) Thiol (FITC-PEG-SH 5000 MW) were all purchased from Sigma-Aldrich (Allentown, PA, USA). Anodic aluminum oxide (AAO) was purchased from InRedox (Longmont, CO, USA), and PBS was purchased from Corning (Corning, NY, USA), ExoQuick TC from System BioSciences (Palo Alto, CA, USA), and 300 Mesh carbon coated TEM grids from Electron Microscopy Sciences (Hatfield, PA, USA). DMEM High Glucose, Fetal Bovine Serum (FBS), Primocin, and 4-(2-hydroxyethyl)-1-piperazineethanesulfonic acid (HEPES) were provided from Fisher Scientific (Waltham, MA, USA).

### 2.2. Synthesis

#### 2.2.1. Ni Magnetic Nanowires (MNWs)

The Ni MNWs were synthesized using a well-known template-assisted electrodeposition [16,17]. Briefly, the electrolyte was composed of 1 M nickel sulfate and 0.5 M boric acid at approximately pH 3. A 7 nm layer of Ti and a 200 nm layer of Cu were successively sputtered on one side of the AAO templates to provide conductivity for electrodeposition. Then, the electrodeposition was performed using a three-electrode apparatus at a constant voltage of −1.0 V. The MNWs were released from the AAO templates using 1 M NaOH and rinsed at least 3× using deionized (DI) water before surface functionalization. For surface functionalization, the Ni MNWs were incubated in 0.5 M NaCl solution containing 1 mg PEG/mg Ni at approximately pH 13 for 24 h at 4 °C and being sonicated occasionally in between. Finally, the Ni MNWs were rinsed using phosphate buffered saline (PBS) at least 3 times to be neutralized before the internalization.

#### 2.2.2. Fe_3_O_4_ Nanorods (NRs)

Fe_3_O_4_ NRs were synthesized by a solvothermal method as reported previously [18,19]. In the synthesis of Fe_3_O_4_ NRs, a mixture of hexadecylamine, oleic acid, and 1-octanol was magnetically stirred at 55 °C to obtain a clear solution. The solution was cooled down to room temperature while still under magnetic stirring, and subsequently, iron pentacarbonyl was added. After stirring for 60 min, the solution mixture was transferred to a 40 mL autoclave with Teflon lining and heated at 200 °C for 6 h. Then, the autoclave was left to cool down to room temperature, and the black precipitate was washed with ethanol. The particles were dispersed in hexane for storing and subsequently dried at room temperature for characterization. For the internalization experiments, the NRs were stained with FITC stain using a 1 mM fluorescein PEG thiol (FITC-PEG-SH 5000 MW).

#### 2.2.3. Magnetosomes (MGs)

MGs extracted from *Magnetospirillum gryphiswaldense* MSR-1 (DMSZ 6631) were employed in this work. The bacteria were cultured in a standard medium as described elsewhere [20]. The medium was enriched with iron by adding 100 μM of Fe(III)-citrate. Cultures were carried out in three-fourths filled 1 L bottles at 28 °C without shaking for 120 h, when well-formed MGs are observed. Then, bacteria were collected by centrifugation at 8000× *g* for 15 min, suspended in 20 mM HEPES−4 mM EDTA (pH 7.4), and disrupted using a French press at 1.4 kbar. The lysate was centrifuged at 600× *g* for 5 min to remove cell debris. Then, the MGs were isolated from the supernatant using a magnetic rack and rinsed 10 times with 10 mM HEPES−200 mM NaCl (pH 7.4). Finally, the isolated MGs were dispersed in deionized water (pH 7.4), sterilized in autoclave (115 °C, 15 min), and stored at 4 °C.

### 2.3. Cell Culture

Canine osteosarcoma cell lines (OSCA-8: derived from a tumor sample taken from the left shoulder of a two-year-old male Rottweiler dog diagnosed with osteosarcoma (Comprehensive Cancer Center, University of Minnesota), human osteosarcoma cell line (U2OS: American Type Culture Collection), human breast cancer cell line (MCF-7: American Type Culture Collection), and human pulmonary adenocarcinoma cell line (A-549: American Type Culture Collection) were cultured as described in our previous work [9]. The cells were incubated for approximately 18 h before treatment with magnetic nanostructures. This is necessary for the cells to get adherent. We have observed that adding nanostructures after the cells are adherent resulted in a better internalization.

### 2.4. Transmission Electron Microscopy

We used a FEI Tecnai T12 Electron Microscope to obtain bright-field TEM images of the nanostructures and cell samples, as described in our previous work [9]. To prepare the cell samples, we fixed the cells with a mixture of 2.5% glutaraldehyde and 0.1 M cacodylate. After three times washing with 0.1 M sodium cacodylate, the samples were post-fixed with 1% OsO_4_, dehydrated in a grade series of ethanol and embedded in Epon 812 resin. Then, the samples were sliced to 65 nm thick layers with a Leica UC6 microtome, and placed on TEM grids. The grids were stained with uranyl acetate (UA) and lead citrate.

### 2.5. Magnetic Measurements

We measured the hysteresis loops (magnetization versus field) of the 3 samples using a Quantum Designs MPMS2 cryogenic susceptometer and a Physical Property Measurement System (PPMS) with a Vibrating Sample Magnetometer (VSM). For sample preparation, we placed 1 mg of sample powder in gel caps and pressed them to avoid any physical movement.

### 2.6. Scanning Electron Microscopy

After the OSCA-8 cells + 30 µg Ni MNWs sample were fixed and dehydrated, we put a droplet of the sample on a TEM grid and placed the TEM grid on an SEM stub. The sample was left to dry for a day, and then we used a Focused Ion Beam (Dual-Beam FIB/SEM) (FIB)—FEI Helios G4 UX for imaging.

### 2.7. Fluorescence Microscopy

We prepared the samples following the instructions discussed in our previous work [9]. In short, we incubated 3 × 10^5^ OSCA-8 cells in a 35 mm glass bottom dish for 18 h. Then, we added the desired amount of nanostructures to the cells (30 μg), which had been coated with PEG and fluorescein isothiocyanate (FITC), and let the cells incubate with them for 48 h. To stain the cells, we used Hoechst 33,342 Solution 20 mM (blue) for the nucleus and CellMask™ Deep Red Plasma Membrane Stain for the membrane. After incubation, we aspirated the medium, washed the cells with PBS, and added 2 mL medium to each glass bottom dish. To obtain the images, we used an Olympus FluoView FV1000 IX2 Inverted Confocal with an FLIM detector in the UMN imaging center.

### 2.8. Isolation of TEX

We first isolated TEX from OSCA-8 cells, which were not treated with magnetic nanostructures to compare the results with TEX isolated from OSCA-8 cells treated with magnetic nanostructures. For this purpose, we employed the ExoQuick-TC + centrifugation method. Then, we incubated the cells with Ni MNWs and isolated the TEX both magnetically and with ExoQuick-TC + centrifugation. In case of Fe_3_O_4_ NRs and MGs, we isolated the TEX only magnetically.

To isolate TEX using ExoQuick-TC + centrifugation, we cultured 3 × 10^6^ cells in a T-150 flask, added 20 mL of exosome-depleted fetal bovine serum (FBS), and incubated the cells for 48 h. Then, we transferred the medium to a 50 mL conical centrifuge tube without disturbing the cells. To remove the bigger debris and dead cells, we centrifuged the medium at 500 g for 10 min and then transferred the medium to another centrifuge tube. After removing all the debris and dead cells in several centrifugation steps, as explained before [9], we added ExoQuick-TC at a ratio of 1:5, and incubated the mixture at 4 °C for 12 h. Then, we centrifuged the mixture, discarded the supernatant, and dispersed the remaining pellet in 500 μL of PBS for NanoSight analysis.

In case of isolating TEX from cells treated with MNWs using ExoQuick-TC + centrifugation, the steps are the same. The only difference is we cultured the cells in the 6-well plate (3 × 10^5^ cells in each well), incubated the cells for 18 h, and then added Ni MNWs to each well. The reason was we could have a better control on dispersing the Ni MNWs in a 6-well plate than in a flask.

For magnetic isolation of TEX, we again cultured 3 × 10^5^ cells in each well of the 6-well plate and incubated the cells for 18 h; then, we added 30 µg of nanostructures to the cells in each well of the 6-well plate. After 48 h, the medium was transferred to glass vials without disturbing the cells, and we placed the vials in a magnetic stand for 15 min. Then, while the vial was still in the magnetic stand, we pipetted out the supernatant without touching the walls of the vials and discarded it. The TEX-containing magnetic nanostructures remained attached to the walls. After that, we removed the vial from the magnetic stand, and for NanoSight measurements we added 500 µL of PBS to the vial and sonicated for 30 s to suspend the TEX. To prepare the TEX for electron microscopy, we fixed and dehydrated them the same as the cells, but the vial was left in the magnetic stand at all the times to avoid losing TEX in the process.

In both cases, after TEX isolation, the cancer cells were discarded by adding 10% bleach to the 6 well plates for 15 min and subsequent aspiration.

### 2.9. Nanoparticle Tracking Analysis

We have used a Nanoparticle Tracking Analyzer (NanoSight) to measure the size distribution of the TEX. NanoSight uses a 400 nm (near UV) laser inside a flow-cell to track the Brownian motion of nanoparticles suspended in the cell and calculate their size distributions. To measure the size distribution of TEX after isolation, we suspended them in PBS. The flow-cell was washed with DI water 3 times before loading the TEX samples. Then, the samples were sonicated for 30 s, and we used a one ml syringe to load the TEX inside the flow-cell. After the flow-cell was filled, we carried out the data analysis to obtain the TEX size distribution. Three measurements per sample were recorded for averaging and error distribution analysis.

## 3. Results and Discussion

### 3.1. Structural and Magnetic Characterization of Nanostructures

**Figure 1:**
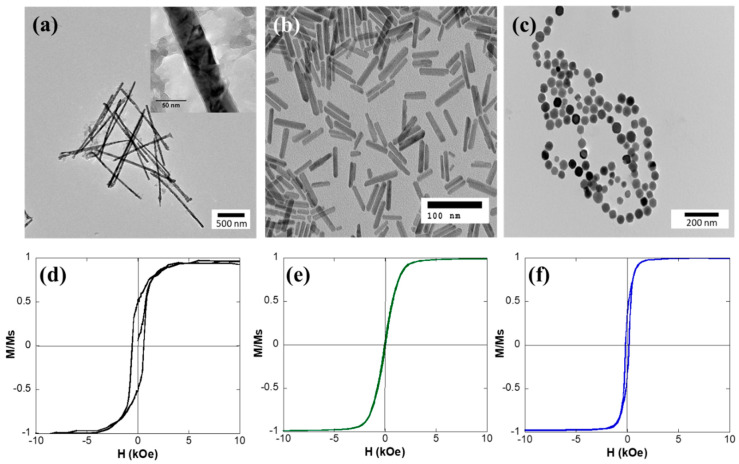
TEM images of (**a**) Ni MNWs, (**b**) Fe_3_O_4_ NRs, and (**c**) MGs. The inset to (**a**) shows a zoomed picture of an Ni MNW. M-H loops measured at 300 K for (**d**) Ni MNWs, (**e**) Fe_3_O_4_ nanorods, and (**f**) Fe_3_O_4_ magnetosomes.

Figure 1a–c presents representative TEM images of the Ni MNWs, the Fe_3_O_4_ NRs, and Fe_3_O_4_ MGs. The MNWs present as long wires, occasionally fragmented into smaller pieces, and with a certain amount of surface roughness. On the other hand, the NRs and MGs exhibit well-defined morphologies, which are rod-like in the case of NRs and cube-octahedral in the case of MGs, as seen in the TEM images. Their corresponding dimensions and composition are included in Table 1. As can be seen, the MNWs are overall much larger than the NRs and the MGs. These MNWs present nanometric width (approximately 40 nm) but micrometric length (approximately 1.5 µm). On the other hand, both the NRs and MGs are truly in the nanometric range, with average sizes of 41 × 7 nm and 45 nm, respectively. In addition, the size distribution of the NRs and the MGs is relatively narrow (relative standard deviation ≤ 15%), while for the MNWs, their width remains mostly constant thanks to the use of the AAO template, but there is some spread in their length distribution. In addition, it must be clarified that for the internalization experiments, both MNWs and NRs were coated with PEG (not shown in the TEM images) to improve their biocompatibility and stability [21]. On the other hand, the MGs already have an innate lipid bilayer coating (approximately 2 nm thick), which confers them with good stability and biocompatibility [22,23].

Concerning the magnetic response, it can be seen (Figure 1d–f) that each sample presents a distinct magnetic behavior. All the M-H loops reach saturation at room temperature by applying fields ≥ 2 kOe. This is important because the maximum field obtained with the typical magnets employed for magnetic isolation is around 5–10 kOe, depending on the distance to the surface of the magnet. Therefore, magnetic nanostructures with lower saturating fields values will be, in principle, more easily attracted by the magnet and hence more easily isolated. Along these lines, another important factor to improve the magnetic separation is the saturation magnetization value, *M*_s._ The experimentally obtained *M*_s_ value of the MNWs is lower than those of the NRs and MGs, but in all the cases, the recorded *M*_s_ values are close to the expected bulk values (Ni bulk, 54.5 emu/g, and Fe_3_O_4_ bulk, 92 emu/g). The most notable differences in the magnetic response can be found in the coercive field, *H*_c_, and normalized remanence, *M*_r_/*M*_s_, values (see Table 1). The MNWs possess high coercive field (535 Oe) and normalized remanence values (0.48). A similar behavior has been described before for the electrodeposited Ni MNWs [24], which was associated with their high aspect ratio, giving rise to a dominant shape anisotropy and to a predominant magnetization reversal through domain wall propagation. On the other hand, the NRs and MGs are both single domain and exhibit lower *H*_c_ values due to their reduced shape anisotropy in comparison to the MNWs. The MGs possess the typical *H*_c_ (175 Oe) and *M*_r_/*M*_s_ (0.36) values expected for low interacting single domain faceted Fe_3_O_4_ nanoparticles [25], while in the case of NRs, both values are appreciably smaller (*H*_c_ = 50 Oe, and *M*_r_/*M*_s_ = 0.04). A similar reduction in *H*_c_ and *M*_r_/*M*_s_ has been reported before for other nanoparticles, which has been attributed to the effect of interparticle interactions [18,26]. Interactions between magnetic nanostructures can be problematic in biomedical applications, since they can make the nanoparticles agglomerate, hindering their magnetic response and their medical implementation [27]. As depicted in the TEM images, due to the effect of interactions, the NRs tend to form clusters, while the MGs tend to form chain and ring structures, as typically reported in the literature [28]. Therefore, even if the dimensions of the individual MGs and NRs are much smaller than the MNWs, they can form larger clusters and agglomerated structures of the order of hundreds of nm.

### 3.2. Internalization of Nanostructures in Cancer Cells

**Figure 2:**
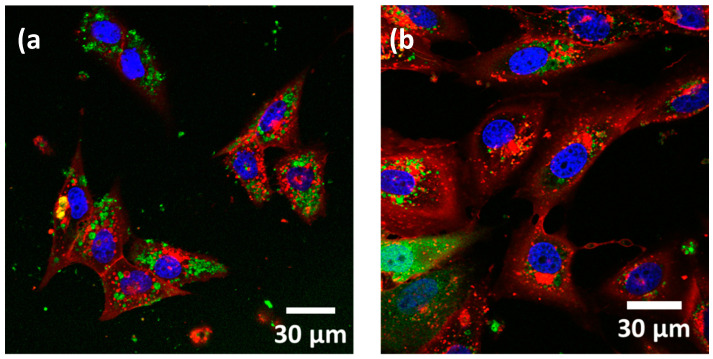
Fluorescence images of (**a**) Ni MNWs and (**b**) Fe_3_O_4_ NRs inside OSCA-8 cells.

Once the three types of nanostructures have been characterized, we carried out the experiments to test their use for magnetic isolation of TEX. The first step was to analyze their internalization by cancer cells. For this, we have worked with an initial concentration of 30 μg of nanostructures per 3 × 10^5^ cells for all the samples (incubated for 48 h), following the results we obtained in our previous work [9]. An initial qualitative assessment of the internalization of these nanostructures by OSCA-8 cells was carried out by using Fluorescence Microscopy (Figure 2). As depicted, most of these nanostructures were apparently located inside the cells, more precisely, within the membrane-bound area, suggesting successful internalization.

**Figure 3:**
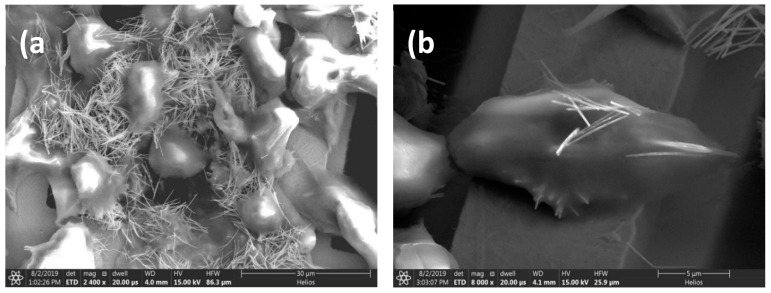
SEM images of Ni MNWs inside OSCA-8 cells with different magnifications: (**a**) image of several cells showcasing the presence of non-internalized MNWs, and (**b**) image of a single cell with internalized MNWs.

Since Fluorescence Microscopy offers a 2D qualitative image of the internalization of the nanostructures, we have also employed alternative techniques to enable a better depiction of the internalized nanostructures. In particular, Scanning Electron Microscopy (SEM) has provided us with images of the Ni MNWs 48 h after being fed to the cancer cells (Figure 3). In these SEM images, many MNWs appear to be located outside the cells (Figure 3a), even after several steps of washing and dehydration with PBS and ethanol. This indicates that a large portion of these MNWs are most likely adhered to the cell surface but not internalized, even 48 h after they were released in the cancer cell medium. No sign of degradation or similar effect is observed in these MNWs. However, SEM images also show plenty of cells with MNWs internalized, as depicted in Figure 3b. Unfortunately, our SEM cannot provide us with a picture of the state of the nanostructures inside the cells. Therefore, in order to have a better insight into how cells internalize these nanostructures, we made ultrathin sections of the cells and visualized them using Transmission Electron Microscopy (TEM).

TEM allows us not only to visualize the exact location of the nanostructures inside the thinly sliced cancer cells but also to obtain a clear depiction, with nanometric resolution, of the state of these nanostructures once they have been internalized. The state of the nanostructures inside the cancer cells (i.e., agglomeration, degradation, fragmentation, etc.) can play a crucial role in their posterior magnetic isolation once they have been released inside/attached to TEX [9].

**Figure 4:**
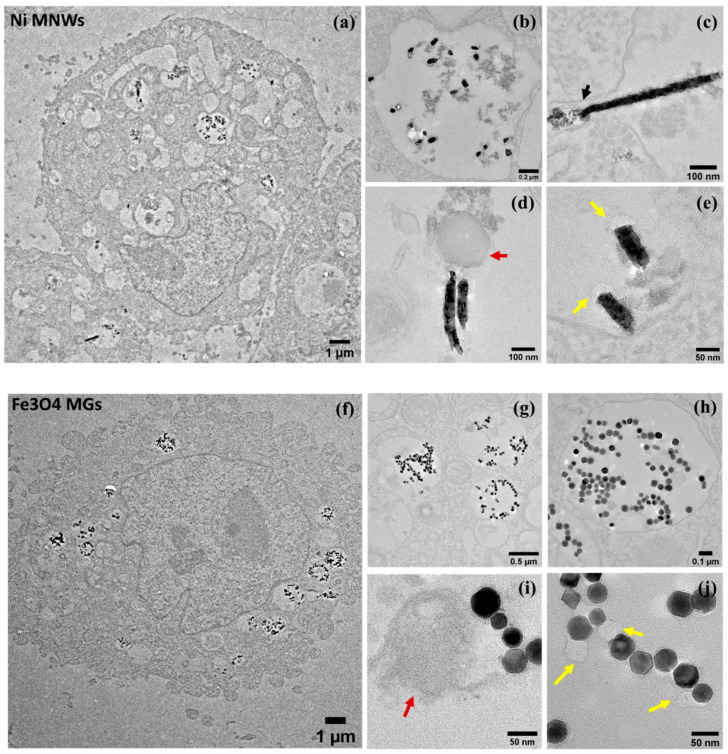
TEM images of Ni MNWs (**a**–**e**) and Fe_3_O_4_ MGs (**f**–**j**) inside OSCA-8 cells. 30 μg per 3 × 10^5^ cells were employed for these studies. Red and yellow arrows have been employed to indicate the presence of different types of intra-endosomal vesicles, as explained in the main text.

In Figure 4, we present the TEM images of Ni MNWs and Fe_3_O_4_ MGs inside OSCA-8 cells. From these TEM images, it can be observed that both Ni MNWs and Fe_3_O_4_ MGs (dark spots) were successfully internalized by the cancer cells. More precisely, as we described in our previous work, these nanostructures are accumulated inside intracellular vesicles called *endosomes* (Figure 4a,b,f,g). These endosomes are membrane-bound vesicles that can package external material (e.g. magnetic nanostructures). These endosomes are key to the biogenesis of exosomes, since in their late stages, they form multivesicular bodies (MVB) that can release these vesicles to the exterior as exosomes. Therefore, if our magnetic nanostructures are compartmentalized inside endosomes, there is chance that they are also incorporated into the exosomes being released outside the cell. The incorporation of nanostructures inside endosomes has been frequently reported in the literature [29,30,31].

Although both MNWs and MGs are internalized inside endosomes, we can observe some clear differences. Qualitatively speaking, the TEM images suggested that the internalization of MGs by cancer cells was slightly higher than for MNWs, but additional experiments would be needed to confirm this. We also tried to assess the effect of increasing the initial concentration of nanostructures on their internalization (see Supporting Information), and in general, we observed an increase in the number of internalized nanostructures per endosome (Figure S1).

On the other hand, for Ni MNWs, in most of the TEM images, only small fragments of the MNWs can be observed, with typical sizes between 50 and 200 nm (Figure 4b–e). Moreover, many of these fragments present, to some extent, clear signs of surface degradation (Figure 4c–e). Similar results have been reported in the literature [9,31,32,33]. This fragmentation/degradation effect has been related to the acidic environment and the different enzymes present inside late endosomes, especially after fusing with lysosomes. A clear example of this degradation process has been captured in Figure 4c, where the tip of a long MNW (indicated by a black arrow) has been clearly broken and degraded into smaller pieces. No degradation was observed in non-internalized MNWs, as depicted in Figure S2. However, we must point out that some of the fragments observed in these TEM images can also be related to the slicing process performed to prepare the resin-embedded samples for TEM imaging [34]. On the other hand, the MGs were also compartmentalized inside endosomes, but their morphology remained mostly unaffected (Figure 4i,j). Although some surface degradation cannot be discarded, it seems that the MGs are more resilient toward degradation than the MNWs. The stability of MGs inside cancer cells has been reported before [30,35]. It is also notable from these TEM images that inside the endosomes, MGs could form chains and similar arrangements, as we had seen before in Figure 1b, instead of being agglomerated in a dense cluster. This is a good indication that the magnetic properties of the MGs remain mostly intact 48 h after internalization.

More importantly, we have also observed the presence of vesicles attached and/or next to the MNWs and MGs (Figure 4d,e). Most of them were either dark round vesicles with sizes around 120–160 nm, indicated by red arrows in the TEM images, or brighter smaller vesicles around 40–50 nm, indicated by yellow arrows. Similar looking vesicles have been reported in TEM images obtained by different groups and frequently identified with intra-endosomal vesicles that could end up being released as exosomes [13,36,37,38]. Although we cannot discard that some of these vesicles, especially the smaller and brighter ones, can be related to some TEM imaging artifact, it must be noted that as has been recently reported, exosomes can present diverse sizes and morphologies, even for a single cell type [37]. The observation of the attachment of these vesicles to the MNWs and MGs supports the hypothesis that these magnetic nanostructures could end up being released as cargo of TEX.

**Figure 5:**
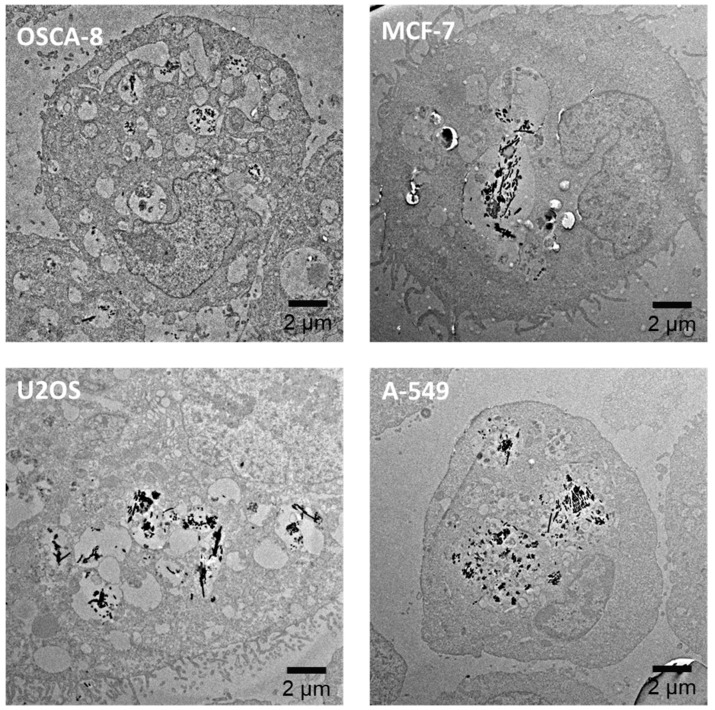
TEM images of Ni MNWs internalized by OSCA-8, MCF-7, U2OS, and A-549 cells.

In addition, as we commented before, it was important to ensure that this magnetic isolation method could be extended to other types of cancer cells, apart from canine osteosarcoma cells. Therefore, we tested 3 other types of cancer cells: (i) human mammary cancer cells (MCF-7), (ii) human osteosarcoma cells (U2OS), and (iii) human lung cancer cells (A-549). In Figure 5, we present representative TEM images of MNWs (same concentration, 30 µg per 3 × 10^5^ cells) internalized by these cells. As depicted, independently of the cell line, internalization and endosomal compartmentalization of the nanostructures was observed. In addition, we have frequently seen intra-endosomal vesicles attached to these nanostructures as in the case of OSCA-8 cells (see Figure S3). These results support the possible applicability of our magnetic isolation method to other types of cancer cells, including human cancer cell lines.

### 3.3. Magnetic Isolation of TEX

Finally, once we ensured that the nanostructures were successfully internalized, we evaluated the feasibility to isolate the TEX released by these cells to the medium using a simple magnetic separation method, as described elsewhere [9]. Basically, we carefully extracted the medium, leaving the cells behind, and transferred it to clean vials. These vials were placed in a magnetic stand for a few minutes, so that the TEX with magnetic nanostructures would be attracted to the magnets and stay attached to the walls of the vial. Then, the rest of the medium was discarded, and after resuspending and sonicating, the sample was ready for further analysis of the isolated TEX. The whole process took less than 1 h, which is faster (and appreciably less expensive) than the standard non-magnetic isolation method we used for comparison, based on a combination of centrifugation and ExoQuick-TC [39].

**Figure 6:**
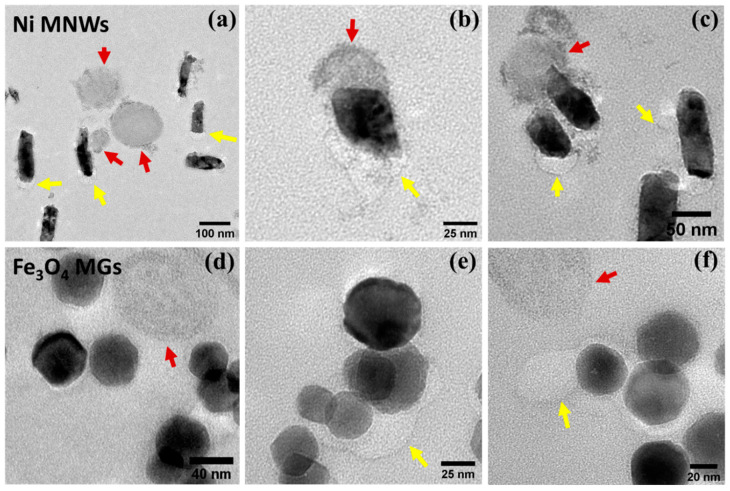
TEM images of vesicles magnetically isolated using (**a**–**c**) Ni MNWs and (**d**–**f**) Fe_3_O_4_ MGs. Red and yellow arrows have been employed to indicate the presence of different types of released vesicles, as explained in the main text.

In order to visually confirm the presence of TEX in the magnetically isolated samples, first, we inspected them under the electron microscope. These samples were prepared in the same way as before: we embedded them in resin, sliced to 65 nm thick layers, and placed on the TEM grids for further inspection. The obtained TEM images are presented in Figure 6. These images depict fragments of MNWs and MGs, together with the different types of vesicles we had observed before inside the endosomal compartments: dark round vesicles (red arrows), and smaller and brighter vesicles (yellow arrows). More images of these isolated vesicles can be found in the Supporting Information (Figure S4). The obtained results clearly confirm that some of these intra-endosomal vesicles have been released by the cancer cells to the exterior with the nanostructures attached, and that our magnetic isolation method is capable of effectively isolating them.

**Figure 7:**
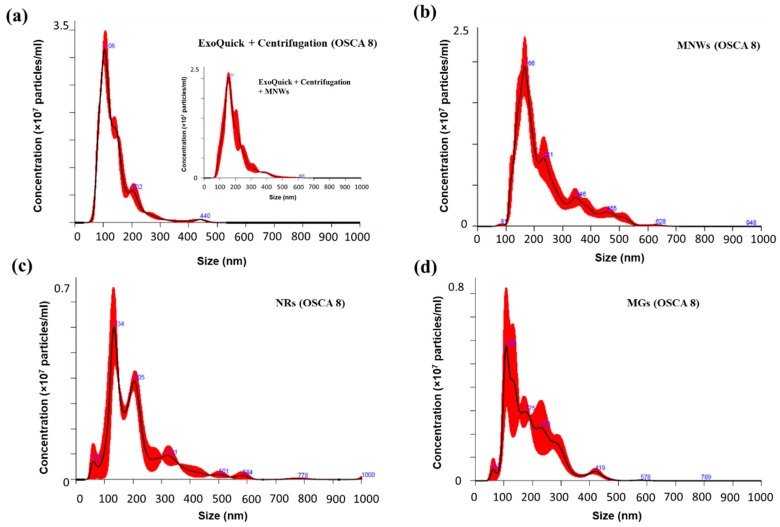
Size distribution (black lines) of (**a**) TEX isolated non-magnetically (using ExoQuick-TC and centrifugation) and magnetically using (**b**) MNWs, (**c**) NRs, and (**d**) MGs. In the inset, the size distribution of TEX isolated non-magnetically after feeding MNWs to the cancer cells is depicted (no magnet was employed). Error bars (in red) indicate ±1 standard error of the mean.

In order to ascertain the nature of these released vesicles and compare them with TEX isolated using conventional methods, we have employed particle tracking using a NanoSight instrument. This is a well-established technique to analyze external vesicles in the range 40–1000 nm, providing particle size distribution profiles and concentration measurements. In addition, it can give us an idea about the purity of the isolated TEX. In Figure 7**,** we present the size distribution of the TEX isolated by using non-magnetic (ExoQuick + centrifugation) and magnetic (MNWs, MGs, and NRs) isolation methods. The main results concerning the size distribution of the isolated TEX, as provided by Nanosight software (v 3.0, Malvern Panalytical, Westborough, MA, USA), are summarized in Table 2. Figure 7a includes the size distribution of the TEX isolated using non-magnetic methods. A main peak is observed around 105 nm, and the size of most of the TEX isolated is between 60 and 210 nm (D10 and D90 values). As can be seen, despite the two smaller peaks around 200 and 440 nm, the size distribution is quite monodispersed. These extra peaks can be in principle associated with the presence of either some non-TEX extracellular vesicles and/or TEX aggregates. Similar results have been reported in the literature for other isolated exosomes [9,40,41]. We also checked that the TEX isolated by using standard methods would not be affected by the presence of MNWs, as indicated in the inset to Figure 7a. Similar results were obtained in both cases.

Now, if we focus on the TEX isolated by MNWs, NRs, and MGs (Figure 7b–d), although the qualitative shape of the size distribution is similar, we can observe some interesting differences. In the three cases, there is also a main peak for the size distribution, but it slightly displaced toward higher sizes: around 130 nm for NRs/MGs and 170 nm for MNWs. We reported a similar behavior in our previous work, and we attributed it to the effect of the attached magnetic nanostructure. This would explain why the peak for the TEX isolated by MNWs fragments, which are larger than NRs and MGs, is displaced toward even higher values (see Table 2). On the other hand, the size distributions obtained for magnetically isolated TEX are also wider and more polydisperse, reaching higher maximum values. These data suggest again that those TEX and extracellular vesicles isolated using our magnetic methods present higher agglomeration than using non-magnetic methods. This polydispersity effect is more noticeable in the case of NRs and MGs than in the case of MNWs. We observed in our TEM images that the NRs and MGs tend to form arrangements and clusters of several nanoparticles, and this could explain the presence of these additional peaks in the size distributions. In addition, it is also relevant to remark that although the concentration of TEX isolated with non-magnetic methods and MNWs is nearly the same (approximately 2.4 × 10^9^ particles/mL), and this value decreases for the TEX isolated with NRs/MGs (approximately 0.7 × 10^9^ particles/mL). Therefore, these results corroborate that our magnetic isolation method can be extended to other magnetic nanostructures apart from MNWs as originally devised, providing in all cases high TEX yields, which are comparable to those obtained through non-magnetic isolation, in a cheaper and faster way. However, it seems that, overall, MNWs provide higher and more monodisperse TEX yields. The reason behind this could be related to, as we pointed out before, the apparently higher internalization of MNWs inside the cells in comparison to MGs and NRs, and/or agglomeration effects.

Finally, we can compare these results with those obtained in recent works using similar methods. For example, in [36], Lim et al. described the use of antibody cocktail-conjugated magnetic nanowires to isolate TEX from plasma of lung cancer patients. They obtained higher concentrations of isolated TEX (approximately 6.3 × 10^9^ particles/mL), but these were more polydisperse, especially compared to our results for TEX isolated with MNWs and ExoQuick. In [42], Belsare et al. showed that magnetic nanoparticles decorated with a CD63-specific DNA aptamer could target and isolate TEX from pancreatic cancer cell lines. They obtained a size distribution with 3 peaks (at 127, 164 and 227 nm), similar to the ones we measured, and proved that the presence of nanoparticles increased the average size of the TEX distribution, as we have also observed. On the other hand, in [43], Nakai et al. used magnetic beads functionalized with a binding protein (Tim4) to purify extracellular vesicles, including TEX, isolated from K562 human leukemia cell lines. They obtained a well-defined single peak in their size distribution, around 106 nm, and demonstrated that using binding proteins and additional purification processes can appreciably improve the purity of the isolated extracellular vesicles in comparison with conventional methods. This can be a next step for improving the quality of the TEX isolated using our magnetic isolation method.

## 4. Conclusions

We have tested the viability of the magnetic isolation method using magnetic nanostructures with different shape, size, and composition, including Ni MNWs, and Fe_3_O_4_ NRs and MGs. We internalized these nanostructures inside OSCA-8 cells. In all cases, the internalized magnetic nanostructures were embedded into endosomal compartments. While the MNWs presented evidence of degradation, the MGs remained mostly intact, indicating that this kind of magnetic nanostructures is more stable and appropriate for repeatable/long-term experiments. After carrying out the magnetic isolation procedure, the nanoparticle tracking analysis results indicated that our magnetic isolation method can make use of other types of magnetic nanostructures apart from MNWs, as originally devised. In all the cases, we obtained TEX yields comparable to those measured through standard non-magnetic methods. Although MNWs exhibited higher degradation and lower magnetization than the NRs and MGs, in the end, they gave higher and more monodispersed TEX yields. This suggests that the high internalization of magnetic nanostructures inside the cells and low agglomeration are key parameters to improving the TEX yield and purity. We expect that the results obtained with Fe_3_O_4_-based magnetic nanostructures (NRs and MGs) can be improved by carrying out additional surface functionalization. We have also shown that MNWs were also internalized by other types of cancer cells, including human cancer cell lines, indicating that the magnetic isolation method can in principle be extended to other types of tumors. In addition, although the magnetic isolation method described here is mainly applicable to vertebrate cells, further investigation in other types of cells, such as lower eukaryotes, could be interesting for future work. Finally, for the implementation of this method in liquid biopsies for cancer detection and prognosis, the nanoparticles should be properly functionalized with targeting ligands so that they exclusively attach to the cancer cells.

## 5. Patents

The magnetic isolation method is covered in published US patent application no. 16/151206 “Nanowire Characterization and Identification”.

## Figures and Tables

**Table 1:** Dimensions, composition, coercivity (*H*_c_), normalized remanence (*M*_r_/*M*_s_), and saturation magnetization (*M*_s_) of Ni magnetic nanowires (MNWs), Fe_3_O_4_ nanorods (NRs), and magnetosomes (MGs) measured at 300 K. The error corresponds with the standard deviation of the size distributions.

Samples	Dimensions (nm)	Composition	*H*_c_ (Oe)	*M*_r_/*M*_s_	*M*_s_ (emu/g)
MNWs	1500(300) × 40(3)	Ni	535	0.48	47
NRs	41(4) × 7(1)	Fe_3_O_4_	50	0.04	86
MGs	45(3)	Fe_3_O_4_	175	0.36	90

**Table 2:** Concentration, mean, mode, and diameters corresponding to 10% (D10) and (90%) cumulative undersize particle size distribution.

	Concentration (10^9^ Particles/mL)	Mean (nm)	Mode (nm)	D10 (nm)	D90 (nm)
ExoQuick + centrifuge	2.43(6)	141(3)	104(3)	61(5)	211(7)
MNWs	2.3(2)	233(3)	167(9)	135(4)	390(10)
NRs	0.67(4)	210(20)	134(2)	120(15)	360(23)
MGs	0.7(1)	190(12)	132(24)	110(10)	300(15)

## Supplementary Materials

The following are available online at https://www.mdpi.com/2079-4991/10/9/1662/s1, Figure S1: TEM images of internalized MNWs and MGs in OSCA-8 cells, Figure S2: TEM images of non-internalized MNWs, Figure S3: TEM images of internalized MNWs and MGs, and Figure S4: TEM images of Magnetically Isolated TEX.

## References

[1] Vlassov A.V., Magdaleno S., Setterquist R., Conrad R. (2012). Exosomes: Current knowledge of their composition, biological functions, and diagnostic and therapeutic potentials. Biochim. Biophys. Acta BBA Gen. Subj..

[2] Lee Y., Andaloussi S.E., Wood M.J. (2012). Exosomes and microvesicles: Extracellular vesicles for genetic information transfer and gene therapy. Hum. Mol. Genet..

[3] Stanley S. (2014). Biological nanoparticles and their influence on organisms. Curr. Opin. Biotechnol..

[4] Huang T., Deng C.-X. (2019). Current Progresses of Exosomes as Cancer Diagnostic and Prognostic Biomarkers. Int. J. Biol. Sci..

[5] (2018). Cancer Fact Sheet. https://www.who.int/news-room/fact-sheets/detail/cancer.

[6] Soung Y.H., Ford S., Zhang V., Chung J. (2017). Exosomes in Cancer Diagnostics. Cancers.

[7] Taylor D.D., Gercel-Taylor C. (2008). MicroRNA signatures of tumor-derived exosomes as diagnostic biomarkers of ovarian cancer. Gynecol. Oncol..

[8] Li P., Kaslan M., Lee S.H., Yao J., Gao Z. (2017). Progress in Exosome Isolation Techniques. Theranostics.

[9] Nemati Z., Um J., Kouhpanji M.R.Z., Zhou F., Gage T., Shore D., Makielski K., Donnelly A., Alonso J. (2020). Magnetic Isolation of Cancer-Derived Exosomes Using Fe/Au Magnetic Nanowires. ACS Appl. Nano Mater..

[10] Nemati Z., Alonso J., Rodrigo I., Das R., Garaio E., Garcia J.A., Orue I., Phan M.-H., Srikanth H. (2018). Improving the Heating Efficiency of Iron Oxide Nanoparticles by Tuning Their Shape and Size. J. Phys. Chem. C.

[11] Xiang Z., Yang X., Xu J., Lai W., Wang Z., Hu Z., Tian J., Geng L., Fang Q. (2017). Tumor detection using magnetosome nanoparticles functionalized with a newly screened EGFR/HER2 targeting peptide. Biomaterials.

[12] Vargas G., Cypriano J., Correa T., Leão P., Bazylinski D.A., Abreu F. (2018). Applications of Magnetotactic Bacteria, Magnetosomes and Magnetosome Crystals in Biotechnology and Nanotechnology: Mini-Review. Molecules.

[13] Qi H., Liu C., Long L., Ren Y., Zhang S., Chang X., Qian X., Jia H., Zhao J., Sun J. (2016). Blood Exosomes Endowed with Magnetic and Targeting Properties for Cancer Therapy. ACS Nano.

[14] Thakur P., Chahar D., Taneja S., Bhalla N., Thakur A. (2020). A review on MnZn ferrites: Synthesis, characterization and applications. Ceram. Int..

[15] Carr B., Hole P., Malloy A., Nelson P., Wright M., Smith J., Park M. (2009). Applications of nanoparticle tracking analysis in nanoparticle research—A mini-review. Eur. J. Parenter. Pharm. Sci..

[16] Kouhpanji M.R.Z., Stadler B.J.H. (2020). Projection method as a probe for multiplexing/demultiplexing of magnetically enriched biological tissues. RSC Adv..

[17] Kouhpanji M.R.Z., Um J., Stadler B.J.H. (2020). Demultiplexing of Magnetic Nanowires with Overlapping Signatures for Tagged Biological Species. ACS Appl. Nano Mater..

[18] Das R., Alonso J., Porshokouh Z.N., Kalappattil V., Torres D., Phan M.-H., Garaio E., Garcia J.A., Llamazares J.S., Srikanth H. (2016). Tunable High Aspect Ratio Iron Oxide Nanorods for Enhanced Hyperthermia. J. Phys. Chem. C.

[19] Chandra S., Das R., Kalappattil V., Eggers T., Harnagea C., Nechache R., Phan M.-H., Rosei F., Srikanth H. (2017). Epitaxial magnetite nanorods with enhanced room temperature magnetic anisotropy. Nanoscale.

[20] Fdez-Gubieda M.L., Muela A., Alonso J., Garcia-Prieto A., Olivi L., Fernández-Pacheco R., Barandiarán J.M. (2013). Magnetite Biomineralization in Magnetospirillum gryphiswaldense: Time-Resolved Magnetic and Structural Studies. ACS Nano.

[21] Gupta A.K., Gupta M. (2005). Synthesis and surface engineering of iron oxide nanoparticles for biomedical applications. Biomaterials.

[22] Alphandéry E. (2014). Applications of Magnetosomes Synthesized by Magnetotactic Bacteria in Medicine. Front. Bioeng. Biotechnol..

[23] Uebe R., Schüler D. (2016). Magnetosome biogenesis in magnetotactic bacteria. Nat. Rev. Microbiol..

[24] Safronov A.P., Stadler B.J.H., Um J., Kouhpanji M.R.Z., Alonso J., Galyas A.G., Kurlyandskaya G.V. (2019). Polyacrylamide Ferrogels with Ni Nanowires. Materials.

[25] Gandia D., Gandarias L., Marcano L., Orue I., Gil-Cartón D., Alonso J., García-Arribas A., Muela A., Fdez-Gubieda M.L. (2020). Elucidating the role of shape anisotropy in faceted magnetic nanoparticles using biogenic magnetosomes as a model. Nanoscale.

[26] Kechrakos D., Trohidou K.N. (1998). Magnetic properties of dipolar interacting single-domain particles. Phys. Rev. B.

[27] Branquinho L.C., Carrião M.S., Costa A.S., Zufelato N., Sousa M.H., Miotto R., Ivkov R., Bakuzis A.F. (2013). Effect of magnetic dipolar interactions on nanoparticle heating efficiency: Implications for cancer hyperthermia. Sci. Rep..

[28] Kiani B., Faivre D., Klumpp S. (2018). Self-organization and stability of magnetosome chains—A simulation study. PLoS ONE.

[29] Sangnier A.P., Prévéral S., Curcio A., Silva A.K.A., Lefèvre C.T., Pignol D., Lalatonne Y., Wilhelm C., Plan A. (2018). Targeted thermal therapy with genetically engineered magnetite magnetosomes@RGD: Photothermia is far more efficient than magnetic hyperthermia. J. Control. Release.

[30] Cypriano J., Werckmann J., Vargas G., Santos A.L.D., Silva K.T., Leão P., Almeida F.P., Bazylinski D.A., Farina M., Lins U. (2019). Uptake and persistence of bacterial magnetite magnetosomes in a mammalian cell line: Implications for medical and biotechnological applications. PLoS ONE.

[31] Safi M., Yan M., Guedeau-Boudeville M.-A., Conjeaud H., Garnier-Thibaud V., Boggetto N., Baeza-Squiban A., Niedergang F., Averbeck D., Berret J.-F. (2011). Interactions between Magnetic Nanowires and Living Cells: Uptake, Toxicity, and Degradation. ACS Nano.

[32] Foroozandeh P., Aziz A.A. (2018). Insight into Cellular Uptake and Intracellular Trafficking of Nanoparticles. Nanoscale Res. Lett..

[33] Mazuel F., Espinosa A., Luciani N., Reffay M., Le Borgne R., Motte L., Desboeufs K., Michel A., Pellegrino T., Lalatonne Y. (2016). Massive Intracellular Biodegradation of Iron Oxide Nanoparticles Evidenced Magnetically at Single-Endosome and Tissue Levels. ACS Nano.

[34] Song M.-M., Song W.-J., Bi H., Wang J., Wu W.-L., Sun J., Yu M. (2010). Cytotoxicity and cellular uptake of iron nanowires. Biomaterials.

[35] Alphandéry E., Idbaih A., Adam C., Delattre J.-Y., Schmitt C., Gazeau F., Guyot F., Chebbi I. (2019). Biodegraded magnetosomes with reduced size and heating power maintain a persistent activity against intracranial U87-Luc mouse GBM tumors. J. Nanobiotechnol..

[36] Lim J., Choi M., Lee H., Kim Y.-H., Han J.-Y., Lee E.S., Cho Y. (2019). Direct isolation and characterization of circulating exosomes from biological samples using magnetic nanowires. J. Nanobiotechnol..

[37] Zabeo D., Cvjetkovic A., Lässer C., Schorb M., Lötvall J., Höög J.L., Lässer C., Lötvall J., Höög J.L. (2017). Exosomes purified from a single cell type have diverse morphology. J. Extracell. Vesicles.

[38] Choi H., Mun J.Y. (2017). Structural Analysis of Exosomes Using Different Types of Electron Microscopy. Appl. Microsc..

[39] ExoQuick Overview. System Biosciences Inc. https://www.systembio.com/microrna-research/exoquick-exosomes/overview.

[40] Measuring Size and Concentration of Exosomes Using Nanoparticle Tracking Analysis (NTA). https://www.azonano.com/article.aspx?ArticleID=3944.

[41] Kharmate G., Hosseini-Beheshti E., Caradec J., Chin M.Y., Guns E.S.T. (2016). Epidermal growth factor receptor in prostate cancer derived exosomes. PLoS ONE.

[42] Belsare S. (2017). Targeting and Enrichment of Exosomes Using Magnetic Nanoparticles. Master’s Thesis.

[43] Nakai W., Yoshida T., Diez D., Miyatake Y., Nishibu T., Imawaka N., Naruse K., Sadamura Y., Hanayama R. (2016). A novel affinity-based method for the isolation of highly purified extracellular vesicles. Sci. Rep..

