# Supplementary material for: Isolation of Cancer-Derived Exosomes Using a Variety of Magnetic Nanostructures: From Fe3O4 Nanoparticles to Ni Nanowires
Source: Nanomaterials (Basel). 2020 Aug 25;10(9):1662. doi: 10.3390/nano10091662 (PMC7558559; doi:10.3390/nano10091662)
Supplement: Supplementary file 1 [file nanomaterials-10-01662-s001.pdf]

Supporting Information

Isolation of cancer derived exosomes using a variety of magnetic nanostructures: from Fe_3O_4 nanoparticles to Ni nanowires

Zohreh Nemati [1,2,3,*], Mohammad Reza Zamani Kouhpanji [1,4], Fang Zhou [5], Raja Das [6,7], Kelly Makielski [2,3,8], Joseph Um [1], Manh-Huong Phan [9], Alicia Muela [10,11], Mª Luisa Fdez-Gubieda [10,12], Rhonda R. Franklin [1], Bethanie J.H. Stadler [1,13], Jaime F. Modiano [2,3,8,14,15] and Javier Alonso [16,*]

1 Department of Electrical and Computer Engineering, University of Minnesota, Minneapolis, MN 55455, USA; zaman022@umn.edu (M.R.Z.K.); umxxx023@umn.edu (J.U.); rfrank01@umn.edu (R.R.F.); stadler@umn.edu (B.J.H.S.)
2 Animal Cancer Care and Research Program, University of Minnesota, Saint Paul, MN 55108, USA; makie001@umn.edu (K.M.); modiano@umn.edu (J.F.M.)
3 Masonic Cancer Research Center, University of Minnesota, Minneapolis, MN 55455, USA
4 Department of Biomedical Engineering, University of Minnesota, Minneapolis, MN 55455, USA
5 Shepherd Labs, University of Minnesota, Minneapolis, MN 55455, USA; zhoux341@umn.edu
6 Faculty of Materials Science and Engineering and Phenikaa Institute for Advanced Study (PIAS), Phenikaa University, Hanoi 10000, Vietnam; raja@phenikaa-uni.edu.vn
7 Phenikaa Research and Technology Institute (PRATI), A & A Green Phoenix Group, 167 Hoang Ngan, Hanoi 10000, Vietnam
8 Department of Veterinary Clinical Sciences, College of Veterinary Medicine, University of Minnesota, Saint Paul, MN 55108, USA
9 Department of Physics, University of South Florida, Tampa, FL 33620, USA; phanm@usf.edu
10 BCMaterials, Basque Center for Materials, Applications and Nanostructures, UPV/EHU Science Park, 48940 Leioa, Spain; alicia.muela@ehu.eus (A.M.); malu.gubieda@ehu.eus (M.L.F.-G.)
11 Department of Immunology, Microbiology, and Parasitology, University of Basque Country (UPV/EHU), 48940 Leioa, Spain
12 Department of Electricity and Electronics, University of Basque Country (UPV/EHU), 48940 Leioa, Spain
13 Department of Chemical Engineering and Materials Science, University of Minnesota, Minneapolis, MN 55455, USA
14 Center for Immunology, University of Minnesota, Minneapolis, MN 55455, USA
15 Stem Cell Institute, University of Minnesota, Minneapolis, MN 55455, USA
16 Department CITIMAC, University of Cantabria (UC), 39005 Santander, Spain
* Correspondence: Z.N. zohre.nematy@gmail.com; J.A. alonsomasaj@unican.es

TEM images of internalized MNWs and MGs in OSCA-8 cells

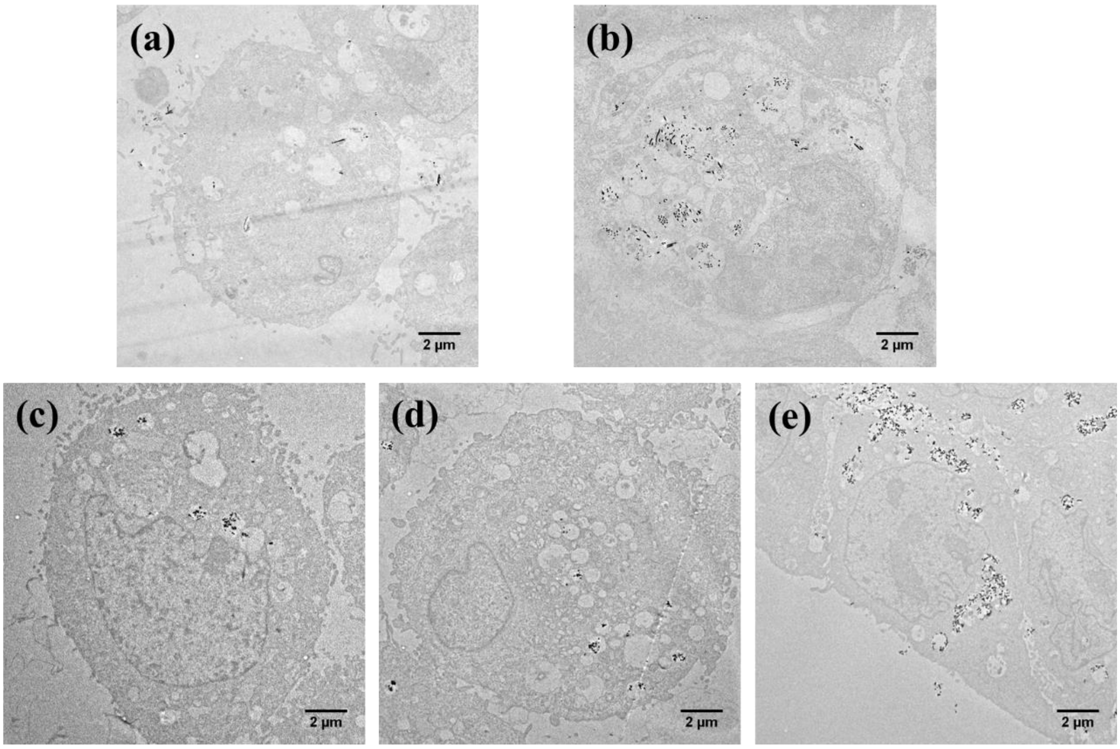

Figure S1: OSCA-8 cells with **(a-b)** 30 and 50 μg of Ni MNWs, and **(c-e)** 30, 50, and 200 μg of MGs per 3 × 10^5 cells.

TEM images of non-internalized MNWs

In order to test if the medium and/or the presence of the cells could also be affecting the MNWs, we took TEM images of MNWs (after 48h) in 2 culture media: normal medium (non-exosome free) and exosome free medium, with and without OSCA-8 cells, after being magnetically isolated. It must be clarified that only the MNWs outside the cells were isolated in this case, no exosome isolation process was carried out. As depicted, in none of the cases we observed degradation of the MNWs, confirming that this only affects those MNWs that have been internalized by the cells.

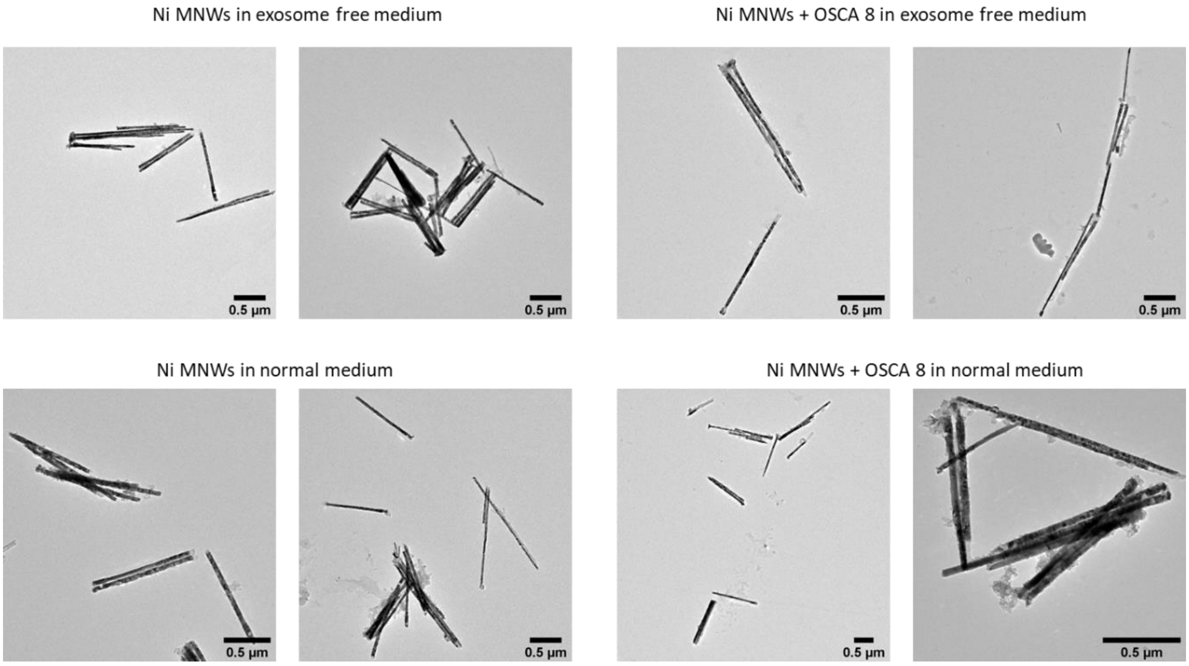

Figure S2: Ni MNWs in different media normal medium (non-exosome free) and exosome free medium, with and without OSCA-8 cells

TEM images of internalized MNWs and MGs

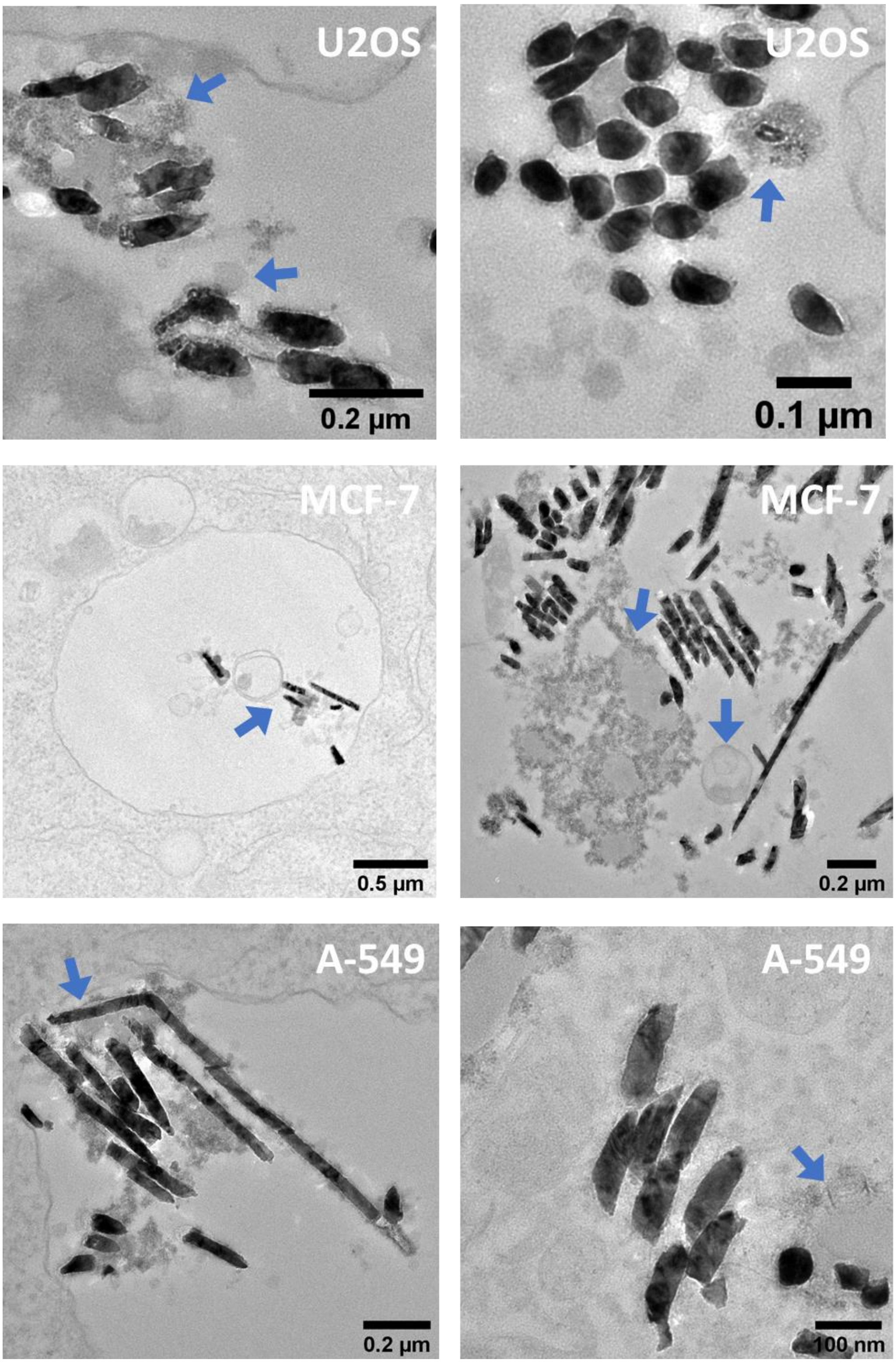

Figure S3: TEM images of MNWs internalized by U2OS, MCF-7, and A-549 cancer cells. Intra-endosomal vesicles have been identified with blue arrows.

TEM images of Magnetically Isolated TEX

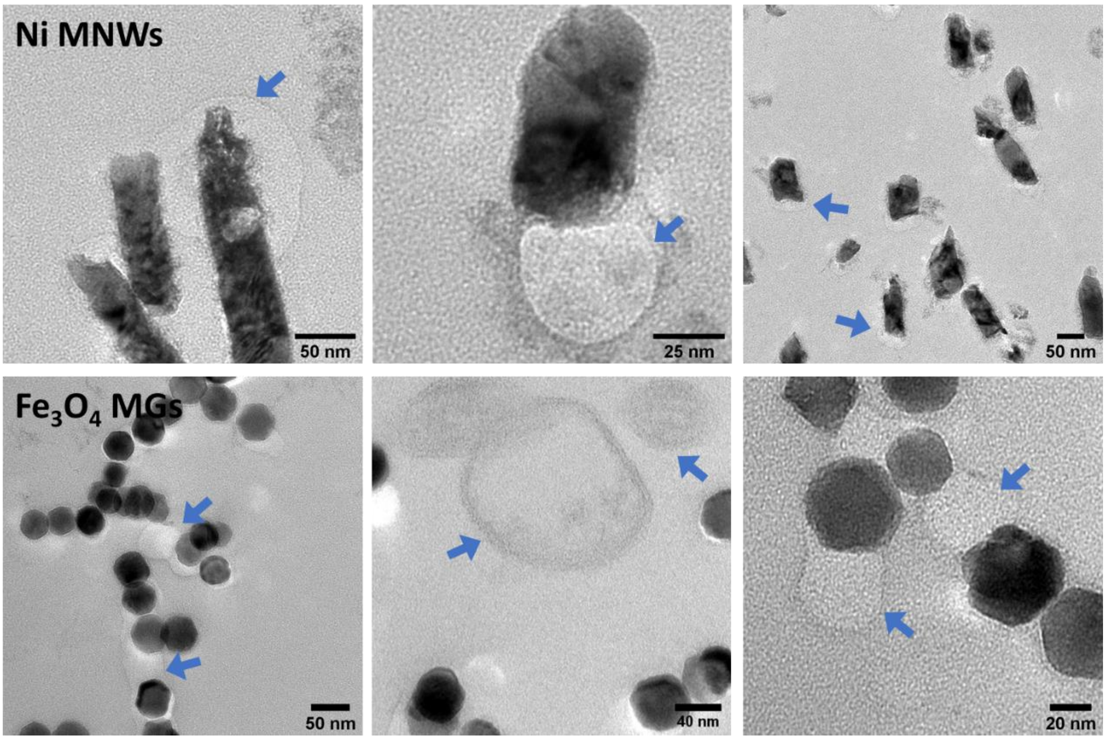

Figure S4: TEM images of TEX isolated by MNWs and MGs. TEX have been identified with blue arrows
